# DRUMS: Disk Repository with Update Management and Select option for high throughput sequencing data

**DOI:** 10.1186/1471-2105-15-38

**Published:** 2014-02-04

**Authors:** Martin Nettling, Nils Thieme, Andreas Both, Ivo Grosse

**Affiliations:** 1 Institute of Computer Science, Martin Luther University, Halle (Saale), Germany; 2 R&D, Unister GmbH, Leipzig, Germany; 3 German Centre for Integrative Biodiversity Research (iDiv) Halle-Jena-Leipzig, Leipzig, Germany

**Keywords:** Database, HERV, SNP, DNA related data, High throughput data

## Abstract

**Background:**

New technologies for analyzing biological samples, like next generation sequencing, are producing a growing amount of data together with quality scores. Moreover, software tools (e.g., for mapping sequence reads), calculating transcription factor binding probabilities, estimating epigenetic modification enriched regions or determining single nucleotide polymorphism increase this amount of position-specific DNA-related data even further. Hence, requesting data becomes challenging and expensive and is often implemented using specialised hardware. In addition, picking specific data as fast as possible becomes increasingly important in many fields of science. The general problem of handling big data sets was addressed by developing specialized databases like HBase, HyperTable or Cassandra. However, these database solutions require also specialized or distributed hardware leading to expensive investments. To the best of our knowledge, there is no database capable of (i) storing billions of position-specific DNA-related records, (ii) performing fast and resource saving requests, and (iii) running on a single standard computer hardware.

**Results:**

Here, we present DRUMS (Disk Repository with Update Management and Select option), satisfying demands (i)-(iii). It tackles the weaknesses of traditional databases while handling position-specific DNA-related data in an efficient manner. DRUMS is capable of storing up to billions of records. Moreover, it focuses on optimizing relating single lookups as range request, which are needed permanently for computations in bioinformatics. To validate the power of DRUMS, we compare it to the widely used MySQL database. The test setting considers two biological data sets. We use standard desktop hardware as test environment.

**Conclusions:**

DRUMS outperforms MySQL in writing and reading records by a factor of two up to a factor of 10000. Furthermore, it can work with significantly larger data sets. Our work focuses on mid-sized data sets up to several billion records without requiring cluster technology. Storing position-specific data is a general problem and the concept we present here is a generalized approach. Hence, it can be easily applied to other fields of bioinformatics.

## Background

With the beginning of the information age in the 90s of the last century, a large set of processes are established to manipulate and analyze data. In particular in the field of bioinformatics, many different workflows produce a growing amount of data. One example are sequencing technologies, which are capable of sequencing an entire human genome in less than a day. Moreover, extensive software suites for analyzing biological data sets exist, e.g. http://galaxy.psu.edu/[1-3]. In addition, it is possible that an analyzing process produces more output data than provided input. For example, the input size of the HERV data set used in this work is about 4 GB. The output of the mapping with BLAST is about 50 GB large. Hence, rapid processes for storing and querying data are needed as it has impact on the general performance of the analytic processes.

### Position-specific DNA related data (psDrd)

In the field of bioinformatics, data related to DNA sequences are of particular importance. Examples are single nucleotide polymorphisms (SNPs) [4], transcription factor binding affinities and probabilities [5,6], and RNAseq data [7,8]. We generalize these types of data by the term position-specific DNA-related data (*psDrd*). A *psDrd* record is an information related to a specific DNA position. *psDrd* records have three characteristics. First, a *psDrd* record *R* can be represented by a key-value pair *R*=(*K*,*V*). The key *K* is composed of the sequence identifier and the position of the associated value *V*. Hence, the key is unique, and records can be easily sorted. Second, *psDrd* records are usually requested by region (e.g., querying for all mutations in a specific gene or looking for transcription factors that are binding near a given position). We call this kind of access *range select*. Third, all *psDrd* of the same kind need the same space to be stored on device, i.e., two different records are represented by the same number of bytes. In contrast, textual annotations are generally of variable length. These three specific properties can be utilized for optimizing data handling of *psDrd*.

### Time- and resource-intensive computations on psDrd

Many biological processes and bioinformatics algorithm have *psDrd* as input or output. This type of data is essential for understanding biological and biochemical processes. Furthermore, diagnostics in medicine for cancer prediction and genetic diseases are using *psDrd*[9-11].

Many activities in bioinformatics focus on analyzing *psDrd*. However, often a file and folder strategy or standard databases like MySQL [12] are used for data management. These approaches are straightforward but not optimized for the intended processing of *psDrd*. In addition, data types used in these tools are expensive and might lead to an exhaustive usage of valuable resources [13]. Both problems lead to resource-intensive requests of *psDrd*. For example, when performing range selects using MySQL, nearly each record in the range must be fetched by a costly random access to the storage. Because of the limits of standard desktop hardware, this might cause a bottleneck during data processing.

### Requirements

The following requirements result from the above mentioned problems: The data management must be usable with standard desktop technology. It must be possible to store billions of data records. Platform independency was defined as an additional requirement (derived from the well-known segmentation of operation systems). Handling massive read requests during analytic processes has to be possible. While optimizing data handling of *psDrd*, the three specific properties from section “*Position-specific DNA related data (psDrd)*” have to be obeyed.

## Implementation

In this section, we first describe a concept called DRUM, on which DRUMS is based. Subsequently, we describe the architecture of DRUMS. Finally, we briefly sketch the implementation of DRUMS in Java considering the three main requirements of handling *psDrd* data sets efficiently.

### DRUM concept

The DRUM (Disk Repository with Update Management) concept [14] allows to store large collections of key-value pairs (KVs). DRUM allows fast bulk inserts without generating duplicate entries. To enable fast processing, incoming *psDrd* records (*K*,*V*) are allocated based on their key *K* to separate buffers *B* in the main memory: $M(K)\to B_{i}$. Those buffers are continuously written to their counterparts on disk (*D*), where they are called *buckets*. If a bucket on disk reaches a predefined size, a synchronisation process with the persistently saved data (on the hard disk) starts. The process is executed in the following way: A disk bucket is entirely read to a disk cache. There it is sorted. Thereafter, a synchronisation is performed by combining each bucket after the other with the corresponding cache. As the records of the disk cache are also sorted, using mergesort is efficient. The synchronisation process is blocking all other processes within DRUM.

The DRUM concept is very suitable for storing *psDrd*. However, requesting data efficiently was never a goal of this approach. Hence, neither single lookups nor range selects have been optimized. Furthermore, when synchronisation is performed, DRUM is not able to receive and cache new *psDrd* records. In the following, we propose an extension of DRUM that addresses these shortcomings.

### Extensions by the DRUMS concept

We extend the DRUM concept by allowing the selection of records by key (*single lookup*) or by range (*range selects*). Within this concept we decoupled I/O-processes from memory processes to avoid blocking single components.

Following the three *psDrd* data properties, the following architecture decisions were made for DRUMS in addition to the DRUM concept: 1) All records are equally sized, so that jumping to the start position of an arbitrary record in the file is possible. Therefore, a sparse index [15] can be applied efficiently, making rapid single selects possible by the following two steps: The sparse index points to a block of records, where the *psDrd* of interest might be found. To finally find the requested record, a binary search is performed. The binary search massively benefits from equally sized records. 2) Records, which are close to each other on DNA are stored close on disk according to their keys. This enables efficient range selects. 3) Records are organized in buckets and chunks, which permits efficient prefiltering of regions of interest within a bucket.

### Architecture of DRUMS

DRUMS is composed of the interacting components described in this section. Before each component is described in detail, we give a high-level overview of the insert and select process of DRUMS.

#### Processes

##### Insert process

**Figure 1:**
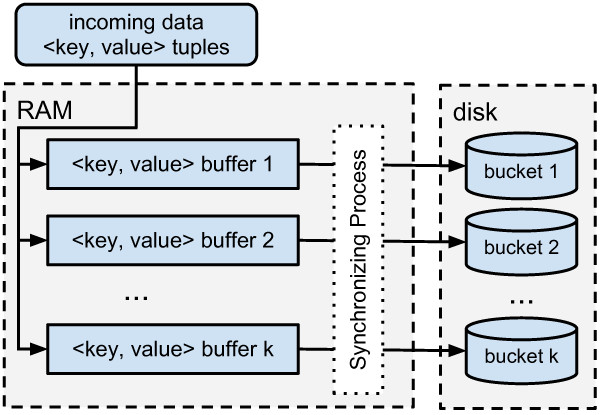
**High level overview of insert process.** Key-value pairs are sent to DRUMS. The incoming records are distributed between *k* buffers (memory buckets), based on their key. If a bucket *B*_*i*_ exceeds a predefined size or memory limitations are reached, a synchronisation process is instantiated.

The high-level overview of the insert process of DRUMS is shown in Figure 1. KV pairs are sent to DRUMS. As in DRUM, the incoming records are already distributed in memory between *n* buffers *B* (called memory buckets). Each bucket *B*_*i*_ in memory has a corresponding bucket *D*_*i*_ on disk. The sizes of the buckets are dynamic. If a bucket *B*_*i*_ exceeds a predefined size or memory limitations are reached, a synchronisation process, consisting of four phases, is started:

1) The bucket *B*_*i*_ is taken and replaced by an empty one. Hence, incoming data can still be buffered. 2) The KV pairs of *B*_*i*_ are sorted by their keys. 3) *B*_*i*_ and *D*_*i*_ are synchronised using mergesort. Already existing records can be updated using state-dependent operations. 4) The merged data is continuously written back to bucket *D*_*i*_. Hence, input data is now saved persistently on the disk.

Note: Step 3 and 4 of the synchronization process are performed chunk-wise, so that optimal read and write performance can be achieved. The optimal chunk-size depends on the used hardware, the size of a single record, the expected data volume, and several parameters in DRUMS. Therefore, it has to be determined empirically.

##### Range select process

**Figure 2:**
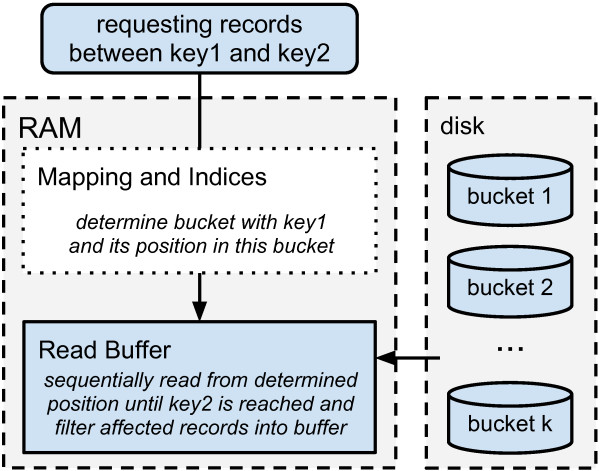
**High level overview of select process.** When a request is sent to DRUMS, four steps are done to read the requested records. 1) The bucket of interest is determined. 2) The correct chunk of the first requested record is identified, using a sparse index. 3) The position of the requested key-value pair is determined. 4) A sequential read is performed until the requested range is completely processed.

Figure 2 shows the high-level overview of the select process. When a request is sent to DRUMS, four steps are performed to read the requested records given by the keys *K*_*S*_ and *K*_*E*_ (start and end of the range). 1) The requested bucket *D*_*i*_ is identified by $M(K)\to D_{i}$. 2) The index of *D*_*i*_ is used for determining the correct chunk *C*_*k*_ of the first requested record *R*_*S*_=(*K*_*S*_,*V*_*S*_). 3) Within *C*_*k*_ a binary search is performed for finding *R*_*S*_. The binary search massively benefits from equally sized records. 4) A sequential read is performed until *K*_*E*_ was found and consequently *R*_*E*_ returned. It might be needed to perform the sequential read over chunk and bucket boundaries.

##### Single select process

A request of a single row (single select) is considered as special case of the range select process where *K*_*S*_=*K*_*E*_. Therefore, it is covered by step 1 to 3.

### Components of DRUMS

#### BucketContainer and its buckets

The BucketContainer is a buffer that is organized in buckets *B* (memory buckets). It manages the distribution of incoming records to the buckets in RAM. As in DRUM, the distribution of the incoming records *R*=(*K*,*V*) to the Buckets *B* is based on a predefined mapping function $M(K)\to B_{i}$.

The BucketContainer is decoupled from any I/O-operation, so that preparing the data for writing can be done in parallel to the I/O-processes. The larger the size of the BucketContainer, the larger are the parts of the data that can be processed sequentially. This increases the performance significantly as sequential I/O-operations are the most efficient on HDDs and SSDs.

#### SyncManager, SyncProcess, and Synchronizer

The SyncManager manages all SyncProcesses. It observes the BucketContainer and verifies the preconditions for the synchronisation of buckets *B* with their counterparts on disk *D*. If these preconditions are fulfilled, the SyncManager instantiates new SyncProcesses. Several SyncProcesses can be run in parallel. In our implementation, a bucket in memory must reach a predefined fill level or age to be synchronized.

A new SyncProcess is always instantiated with the largest bucket in the BucketContainer fulfilling the above mentioned condition. When a new SyncProcess is started, the affected bucket in the BucketContainer is replaced by an empty one. In this way the synchronization process is not blocking further insert operations for this bucket.

The SyncProcess instantiates new Synchronizers. A Synchronizer is in charge of writing data from the bucket *B*_*i*_ in memory to the bucket *D*_*i*_ on disk. All records are sorted in *B*_*i*_ and in *D*_*i*_. Hence, the Synchronizer is capable of using mergesort for synchronizing the records in memory with those on disk.

#### Representation and structure of the data

**Figure 3:**
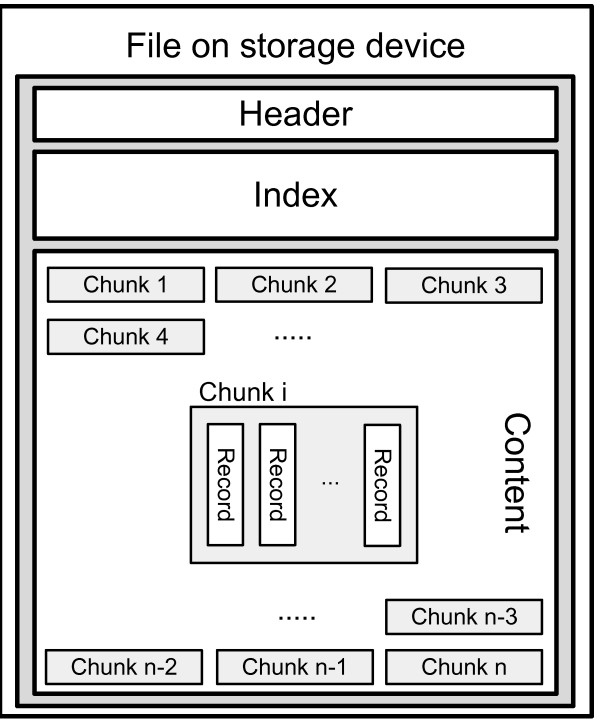
**Structure of a file on storage device.** The file is structured into (i.) a header, (ii.) an index structure and (iii.) the content, containing the records.

Each persistent bucket is represented by a file on a hard disk. The file is structured into two parts (see Figure 3): (i) the header with meta information and the index structure referencing chunks of a predefined size and (ii) the rest of the file used for the records to store, which are organized in chunks. A sparse index [15] is applied as it is memory efficient and takes advantage of the order of *psDrds*.

Whenever a bucket *D* is opened for reading or writing, the header and the index are read into memory. In this way, a rapid access to the required chunks is possible.

The internal representation of a record in a chunk is a sequence of bytes. This sequence is composed of a key-part and a value-part. Each part may consist of several subparts, each of its own data-type (e.g., integer, long, char or even high level data structures like objects). Because of the fact that each record is of equal size, data structures and memory can be easily reused by application of the adaptor and the prototype pattern [16].

### Implementation of DRUMS

DRUMS is build upon Oracle Java 1.6. Therefore, it is platform independent. We developed DRUMS in an atomic thread-based way. All components work asynchronously and are exchangeable. This allows fast adaptations on single subprocesses or exchanging whole components like the Synchronizer.

## Results and discussion

In this section we first give a short introduction into two different *psDrd* sets used for evaluation. Second, we present the results and the evaulation approach considering (i) inserts, (ii) random lookups, and (iii) random range selects.

To prove the superiority of DRUMS in comparison with standard solutions within a desktop environment, we compare it to MySQL which is used widely in the bioinformatics community.

Two different *psDrd* sets are evaluated. The data sets are described below. DRUMS as well as MySQL were tested comparatively using the three measures: (i) - (iii). For all tests a standard desktop computer was used. MySQL as well as DRUMS are limited to use only 2 GB of the available memory. Details can be obtained from Table 1.

**Table 1:** Test system

**Processor**	**Intel Xeon E31225**
	**(4 native cores, no hyperthreading)**
Memory	8 GB
Operation system	Debian 6.0 (Squeeze)
Hard drive	Western digital WD10EALX-759, 32 MB cache

The desktop system which was used for the tests. MySQL as well as DRUMS are limited to use only 2 GB of the available memory.

### Data sets

#### SNP-Data from the 1001 genomes project

The 1001 Genomes Project [17,18] has the goal to understand the resulting of small mutations in different accessions of the reference plant Arabidopsis thaliana. Each accession mainly consists of five attributes: accession identifier, sequence identifier, position on sequence, source base, and target base. We downloaded filtered quality data of the strains sequenced by the Gregor Mendel Institute and the Salk institute on 2012-01-15, containing 251 data sets, with 137,369,902 SNPs. From all files, we extracted the data of the following five columns: accession name, chromosome, position on chromosome reference nucleotide, and mutated nucleotide. For the definitions of the used data types and their configuration (e.g., index properties) used in MySQL and DRUMS see Table 2.

**Table 2:** Data types used for SNP data

**Column**	**MySQL properties**	**DRUMS properties**
Accession name	TINY INT, primary key	1 byte, key part 1
Chromosome	SMALL INT, primary key	2 byte, key part 2
Position on chromosome	INT, primary key	4 byte, key part 3
Reference nucleotide	VARCHAR	1 byte, value part 1
Mutated nucleotide	VARCHAR	1 byte, value part 2

Used data types in MySQL and DRUMS for SNP data. All columns being part of the primary key are indexed.

All data are public available at http://1001genomes.org/datacenter/.

#### HERV data

Human endogenous retroviruses (HERVs) have integrated themselves in the human genome millions of years ago. Because of the high number of existing HERV fragments, they are thought to have a regulatory role. To investigate a possible influence of HERVs, it is needed to locate HERV fragments. Therefore, over 7000 known HERV fragments were blasted against the human genome to find new putative HERV-like regions. In the work of Konstantin Kruse [19] all regions with an E-value less than 1*e*-20 were accepted as putative HERV-like region. This lead to 802,710,938 single records, stored in 20 files with tab-separated data field, with a total size of 50 GB. From these files we used the following seven columns: query id, subject id, query start, query end, subject start, subject end, and E-value. For the definitions of the used data types and their configuration (e.g., index properties) used in MySQL and DRUMS see Table 3.

**Table 3:** Data types used for HERV data

**Column**	**MySQL properties**	**DRUMS properties**
Chromosome	TINY INT, primary key	1 byte, key part 1
Start-position on chromosome	INT, primary key	4 byte, key part 2
End-position on chromosome	INT, primary key	4 byte, key part 3
Start-position on HERV	SMALL INT, primary key	2 byte, key part 4
End-position on HERV	SMALL INT, primary key	2 byte, key part 5
Id of referenced HERV	SMALL INT, primary key	2 byte, key part 6
Strand on chromosome	TINY INT, primary key	1 byte, key part 7
E-value	DOUBLE	4 byte, value part 1

Used data types in MySQL and DRUMS for HERV data. All columns being part of the primary key are indexed.

### Insert performance

DRUMS must be able to store hundreds of millions of records. Because of this, it is needed to evaluate the insert performance.

**Figure 4:**
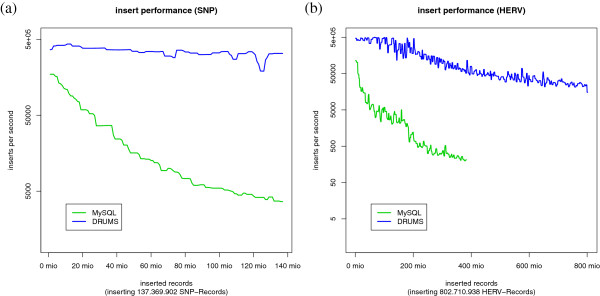
**Insert performance.** The blue line represents DRUMS, the green line represents MySQL. **(a)** Insert performance on SNP-data **(b)** Insert performance on HERV data. Concerning MySQL, it was impossible to insert all 800 million HERV records. DRUMS inserted the complete data set within 4.53 hours.

To estimate the insert performance, we measure the time for inserting 10^6^ records. We obtain 140 time measurements points in case of SNP-Data and 800 for HERV data. Figures 4a and 4b show the insert performance of DRUMS (blue) and MySQL (green). Despite using bulk-requests for inserting the data, it was impossible to insert all 800 million HERV records into the MySQL instance. MySQL inserts about 200 million records in the first week, but Figure 4b shows that the insert performance has dropped to 300 records per second after one week. The insert performance of DRUMS also decreases, but it was able to insert the whole data set within 4.53 hours. At the end of the test, DRUMS was still able to perform more than 20000 inserts per second.

Figure 4a and 4b show that DRUMS has a better insert performance than MySQL on both test datasets. The insert performance of MySQL and of DRUMS decreases with the number of records already inserted. Regarding MySQL one possible explanation is the continuous reorganistation and rewriting of the index.

The insert performance of DRUMS decreases slowly in comparison to MySQL. The reason for this is the decreasing ratio of read- to write-accesses with each round of synchronisation. With other words, DRUMS must read more and more records per new record to write with the growing amount of data already stored on disk. However, DRUMS still inserts more than 20000 records per second at the end of the insert test for HERV data, corresponding to approximately 400 kB per second.

### Performance on random lookups

From the view of bioinformatics, single lookups make no sense in both experiments. However, the performance of single-lookups is a significant indicator for the overall performance and the suitability of the implementation of a tool for handling data sets. Moreover, the test may show how close the measured performance to the theoretical hardware limits of the used standard desktop hardware is. Considering the test environment, it is assumed that a random access would take approximately 20 ms. Hence, if no other disk accesses are done, it would be theoretically possible to read 50 records per second.

**Figure 5:**
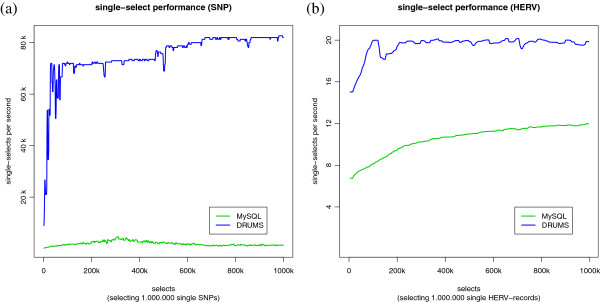
**Random lookups performance.** The blue line represents DRUMS, the green line represents MySQL. **(a)** Random lookup performance on SNP-data. **(b)** Random lookup performance on HERV data.

Figures 5a and 5b show the performance of MySQL and DRUMS, when performing random lookups. Again, DRUMS performs better than MySQL in case of handling our two data sets. Figure 5a implies that DRUMS is able to do 160 times more random lookups than theoretically possible, when accessing SNP data. In comparison, only 20 random lookups per second are performed when accessing HERV data. The reason for this difference are cache structures provided by the operating system and the underlying hardware.

In case of accessing SNP data, the complete data set might be cached by the operating system after approximately 650,000 lookups. Hence, organizing the SNP data as DRUMS structure results in a file size small enough that it could be loaded into memory. Therefore, nearly each request could be answered from the operating systems cache after a warm up. In contrast, the HERV data set is too large to fit into memory, so only a few random lookups could be answered from cache. The increasing performance of MySQL and DRUMS in Figure 5b is also an indication for the use of caches. Figure 5b shows that DRUMS can perform 20 random lookups of theoretically possible 50.

While considering the experimental results of MySQL, the impression is conveyed that the defined index was not used correctly. However, a closer look validates the results as the explicit MySQL index for the SNP table has the size of 2380 MB, which will not fit into the allowed 2 GB of main memory. Hence, even index-based searches in MySQL need several accesses to the hard disk resulting in worse performance. In contrast, the sparse index of each bucket of DRUMS requires just 0.5 MB, which sums up to only 123 MB for all buckets. To find a single record in a chunk, DRUMS performs a binary search. The binary search can be done very efficiently for the reason that all records are of equal size. Because of the reduced demands on the hardware, DRUMS provides a good performance even on very large data sets like HERV.

### Performance on random range selects

As described in the section Background, *psDrd*-records are mostly requested by range. Therefore, the need to benchmark the performance of range requests is obvious.

The request for the SNP-data is as follows: Select all SNPs on chromosome c between position x and y for all ecotypes in the database. To perform the read test for SNP-data, we first randomly generated 10^6^ ranges of length 10^3^ to 10^4^. Second, we request records within those ranges randomly distributed over the whole genome of Arabidopsis thaliana.

Analogously, we generate 10^6^ test requests for the HERV data set with lengths from 10^5^ to 10^6^. Again, we randomly distributed range-requests over the whole human genome. It might be a common task to filter the requested data by value. MySQL provides this functionality by defining the filter condition in the WHERE-clause. To accomplish this in DRUMS, the returned records must be checked iteratively. In this test, we filter the requested HERV records by an E-value less than 10^-20^, 10^-25^, 10^-30^, 10^-35^, 10^-40^, 10^-45^ or 10^-50^, randomly chosen.

**Figure 6:**
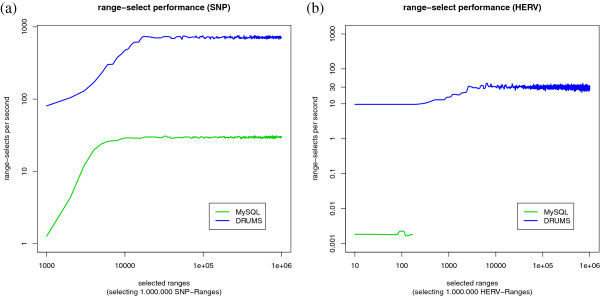
**Range select performance.** The blue line represents DRUMS, the green line represents MySQL. **(a)** Range select performance on SNP-data. **(b)** Range select performance on HERV data. Concerning MySQL, we stopped the test after 26.35 hours. DRUMS read 64 billion records in 9.61 hours.

Figures 6a and 6b show the results of the range select test. Once more, both databases perform much better on the smaller SNP-data set. Besides caching, this time another explanation for this observation is that a range request on the SNP-data contains in average 3 times fewer records than a range request on the HERV data. The performance increases with the number of read records. The performance of DRUMS increases by a factor of 10 and of MySQL by a factor of 26. However, DRUMS performs in average on the SNP-data 24 times faster than MySQL.

Regarding the larger HERV data set, DRUMS is able to perform 30 range-selects per second in average. This is over 15000 times faster than MySQL.

Within the whole test, 64 billion records were read in 9.61 hours. That corresponds to an overall read performance of 35.7 MB per second, filtering included. In contrast, MySQL read 6.6 million records in 26.35 hours, which corresponds to only 1.3 kB per second.

## Conclusions

We defined *psDrd (position-specific DNA related data)* and showed three important properties of this kind of data. The flaws of DRUM were shown, which is already suitable for storing *psDrd*, but not for requesting it efficiently. The article introduces DRUMS, a data management concept optimized to tackle the challenges of dealing with mid-size data sets in form of *psDrd* using standard desktop technology instead of expensive cluster hardware.

An implementation of the DRUMS concept was compared to the widely spread standard database management solution MySQL considering two data sets of the bioinformatics context. On the larger HERV data set, the evaluated DRUMS implementation was 23 times faster inserting all records, two times faster performing random lookups, and 15456 faster performing range requests. Hence, the experiments show that dealing with *psDrd* benefits significantly from the characteristics of the DRUMS concept. Therefore, our main contribution is suggesting this data management concept for increasing the performance during data intensive processes while keeping the hardware investments low.

## Availability and requirements

**Project name:** DRUMS 

**Project home page:**http://mgledi.github.io/DRUMS

**Project home page of examples:**http://github.com/mgledi/BioDRUMS

**Operating system:** Platform independent 

**Programming language:** Java 

**Other requirements:** none 

**License:** GNU GPL v2 

**Any restrictions to use by non-academics:** No specific restrictions.

## Competing interests

The authors declare that they have no competing interests.

## Authors’ contributions

MN and NT developed and tested the Java code. All of the authors contributed to the design of the software architecture. All of the authors read and approved the final version of the manuscript.
